# *ADGRL3 (LPHN3)* variants are associated with a refined phenotype of ADHD in the MTA study

**DOI:** 10.1002/mgg3.230

**Published:** 2016-07-18

**Authors:** Maria T. Acosta, James Swanson, Annamarie Stehli, Brooke S. G. Molina, the MTA Team, Ariel F. Martinez, Mauricio Arcos‐Burgos, Maximilian Muenke

**Affiliations:** ^1^ Medical Genetics BranchNational Human Genome Research InstituteNational Institutes of HealthBethesdaMaryland; ^2^ Department of Pediatric and NeurologyGeorge Washington UniversityChildren's National Medical CenterWashingtonDistrict of Columbia; ^3^ Department of PsychiatryFlorida International UniversityMiamiFlorida; ^4^ Department of PediatricsUniversity of California at IrvineIrvineCalifornia; ^5^ Departments of Psychiatry and PsychologyUniversity of PittsburghPittsburghPennsylvania; ^6^ Genomics and Predictive MedicineGenome Biology DepartmentJohn Curtin School of Medical ResearchANU College of Medicine, Biology and EnvironmentThe Australian National UniversityCanberraACTAustralia

**Keywords:** *ADGRL3*, ADHD, genetics, *LPHN3*, MTA

## Abstract

**Background:**

ADHD is the most common neuropsychiatric condition affecting individuals of all ages. Long‐term outcomes of affected individuals and association with severe comorbidities as SUD or conduct disorders are the main concern. Genetic associations have been extensively described. Multiple studies show that intronic variants harbored in the *ADGRL3 (LPHN3)* gene are associated with ADHD, especially associated with poor outcomes.

**Methods:**

In this study, we evaluated this association in the Multimodal Treatment Study of children with ADHD (MTA), initiated as a 14‐month randomized clinical trial of 579 children diagnosed with DSM‐IV ADHD‐Combined Type (ADHD‐C), that transitioned to a 16‐year prospective observational follow‐up, and 289 classmates added at the 2‐year assessment to serve as a local normative comparison group (LNCG). Diagnostic evaluations at entry were based on the Diagnostic Interview Schedule for Children‐Parent (DISC‐P), which was repeated at several points over the years. For an add‐on genetic study, blood samples were collected from 232 in the MTA group and 139 in the LNCG.

**Results:**

For the 205 MTA participants, 14.6% retained the DISC‐P diagnosis of ADHD‐C in adolescence. For 127 LNCG participants, 88.2% remained undiagnosed by the DISC‐P. We genotyped 15 polymorphic SNP markers harbored in the *ADGRL3* gene, and compared allele frequencies for the 30 cases with continued diagnosis of ADHD‐C in adolescence to the other participants. Replication of the association of rs2345039 *ADGRL3* variant was observed (*P* value = 0.004, FDR corrected = 0.03; Odds ratio = 2.25, upper CI 1.28–3.97).

**Conclusion:**

The detection of susceptibility conferred by *ADGRL3* variants in the extreme phenotype of continued diagnosis of ADHD‐C from childhood to adolescence provides additional support that the association of *ADGRL3* and ADHD is not spurious. Exploring genetic effects in longitudinal cohorts, in which refined, age‐dependent phenotypes are documented, is crucial to understand the natural history of ADHD.

## Introduction

Family, twin, and case–control‐based linkage and association studies indicate that genetics play a crucial role in shaping susceptibility to both ADHD and disruptive behaviors (Arcos‐Burgos et al. 2012; Jain et al. 2012). Using genome wide data, we found evidence of linkage of ADHD to chromosomes 4q13.2, 5q33.3, 8q11.23, 11q22, and 17p11 (Arcos‐Burgos et al. 2004). Furthermore, linkage and association analyses revealed not only cosegregation between ADHD and disruptive behaviors but also the existence of common signals of linkage to several loci (Jain et al. 2007). Reverse genetics allowed us to identify variants in the *adhesion G‐protein‐coupled receptor L3 gene (ADGRL3,* also known as *latrophilin 3* or *LPHN3)* (Accession number NC_000004.12, GRCh38.p2) (OMIM:616417) at 4q13.2 that predisposes to ADHD and disruptive behaviors (Arcos‐Burgos et al. 2010; Acosta et al. 2011; Domene et al. 2011; Martinez et al. 2011; Ribases et al. 2011; Jain et al. 2012). Using cohorts of thousands of individuals from Colombia, Germany, Norway, Spain, and the United States, we found a significant homogeneous genetic effect of *ADGRL3* variants predisposing to ADHD in children, adolescents, and adults, and predicting the response to stimulant medication, (Arcos‐Burgos et al. 2010, 2012; Ribases et al. 2011; Fallgatter et al. 2013). These results were independently replicated in four additional pharmacogenetic studies from Canada, Brazil, and Korea (Choudhry et al. 2012; Labbe et al. 2012; Bruxel et al. 2015; Hwang et al. 2015).

ADGRL3 is a member of the latrophilin subfamily of G‐protein‐coupled receptors (GPCRs) (Martinez et al. 2011). Latrophilin ADGRL1 and ADGRL2 serve as receptors for alpha‐latrotoxin, a component of the venom of the black widow spider (*Latrodectus mactans*) (Martinez et al. 2011). We showed that ADGRL3 is only expressed almost exclusively in the brain and mostly is highly expressed in regions neurophysiologically associated with the pathology of ADHD (Arcos‐Burgos et al. 2010). Recently, the identification of the fibronectin family of leucine‐rich repeat transmembrane protein 3 (FLRT3) was identified as an endogenous postsynaptic ligands for ADGRL3, facilitated the identification of a high‐affinity trans interaction between the ADGRL3 ectodomain with its ligand, FLRT3 (O'Sullivan et al. 2012) and interfering with this interaction reduces excitatory synapse density in cultured neurons and decreases afferent input strength and dendritic spine number in dentate granule cells. This suggests that the ADGRL3 and its ligand‐ FLRT3 partnership plays an important role in glutamatergic synapse development (O'Sullivan et al. 2012). We found that gene ontology pathways involved in axon guidance, regulation of synaptic transmission, and regulation of transmission of nerve impulse, are overrepresented when *ADGRL3* variants associated with ADHD are tested. In agreement with our results, data from a mutant knockout mouse model null for the *Adgrl3,* gene as well as the loss of function of the ortholog *adgrl3.1* during zebrafish development, also support the implication of *ADGRL3* in ADHD pathophysiology (Arcos‐Burgos et al. 2012; Lange et al. 2012; Wallis et al. 2012).

In this study, we explored the association of *ADGRL3* variants with ADHD in the Multimodal Treatment Study of children with ADHD (MTA). The MTA study was initiated as a 14‐month randomized clinical trial of 579 children diagnosed with DSM‐IV ADHD‐Combined Type (ADHD‐C, between 7.0 and 9.9 years of age) that transitioned to a 16‐year prospective observational follow‐up, and 289 classmates added at the 2‐year assessment to serve as a local normative comparison group (LNCG). Outcomes at the end of the randomized clinical trial and at follow‐up in childhood and adolescence have been reported (1999; Jensen et al. 2007; Molina et al. 2013, 2009; Molina and Pelham 2003). Exploring genetic association in longitudinal studies, in which age‐dependent refined phenotypes are ascertained prospectively, is crucial to better understand genetic effects on the natural history of ADHD (Faraone et al. 2006; Franke et al. 2012).

## Material and Methods

### Ethical complience

The MTA study is a cooperative treatment study performed by six independent research teams in collaboration with the Division of Services and Intervention Research, National Institute of Mental Health, and the Office of Special Education Programs, U.S. Department of Education, Washington, DC (The MTA Cooperative Group 1999). Research was conducted in accordance with the ethical guidelines of both local institutional review boards and the National Institutes of Health Office for Protection From Research Risks, Bethesda, MD.

### Subjects

The MTA was designed to evaluate long‐term effects of treatments for ADHD in a 14‐month randomized controlled trial of 579 children assigned to a four treatment groups – medication management, behavior modification, their combination, and treatment as usual in community care. After the 14‐month treatment‐by‐protocol phase, the MTA continued as a naturalistic follow‐up, in which self‐selected use of psychoactive medication was monitored. A LNCG of 289 randomly selected classmates group‐matched for grade and sex was added when the ADHD participants were between 9 and 12 years of age. The outcomes in childhood (14, 24, 36 months after baseline), and adolescence (6 and 8 years after baseline) have been reported (1999; Jensen et al. 2007; Molina et al. 2013, 2009; Molina and Pelham 2003). In the MTA, the participants were diagnosed in childhood based on the DSM‐IV criteria for ADHD subtypes (hyperactive/impulsive, inattentive, and combined) using the Diagnostic Interview Schedule for Children‐Parent Version (DISC‐P), supplemented with teacher report of symptoms. The DISC‐P was administered at entry (in childhood) and at each of the prospective follow‐up assessments, including the 6‐year follow‐up when the participants were between 13.0 and 15.9 years of age in thus in adolescence.

Clinical observations and longitudinal studies document that symptom presence and severity decrease over time in some children with ADHD, resulting in decreased diagnosis with age (Faraone et al. 2006; Franke et al. 2012). In the prospective follow‐up of the MTA, a refined phenotype based on DSM‐IV diagnostic persistence was established by determining the cases that maintained the parent‐reported symptoms of the disorder in adolescence (persistent ADHD‐C). We used this refined phenotype to study the association of the *ADGRL3* genetic variants with ADHD. We hypothesized that those with persistence of the symptoms that qualified them for entry into the study as children would be more likely as adolescents to have *ADGRL3* genetic susceptibility compared to those whose diagnostic subtype shifted or disappeared. This highly vulnerable subgroup may also be more susceptible to poor outcomes as described elsewhere (Arcos‐Burgos et al. 2010).

### Genotyping

Genomic DNA was extracted from whole blood using the QIAamp DNA Blood Maxi Kit (QIAGEN, Germantown, MD). Samples were subjected to whole‐exome genotyping using the Illumina^®^ HumanExome BeadChip‐12v1_A, which covers putative functional exonic variants selected from over 12,000 individuals. The exonic content consists of >250,000 markers representing diverse populations – including European, African, Chinese, and Hispanic individuals – and a range of common conditions, such as type 2 diabetes, cancer, metabolic, and psychiatric disorders. Four open controls per 96‐well‐plate were used for internal quality control, including two positive and two blank controls. Duplicates assessed data quality across all plates. Genotyping from nonmatching duplicates was dropped. Only data generated by SNP assays that were successfully genotyped on at least 90% of samples were analyzed. In addition, we genotyped the *ADGRL3* (Accession number NC_000004.12, GRCh38.p2) susceptibility haplotype variants rs2345039, rs6551665, and rs1947274 previously reported (Arcos‐Burgos et al. 2010), which were not represented in the exome chip. Briefly, genotyping was performed using TaqMan^®^ SNP Genotyping Assays (Thermo Fisher Scientific, Grand Island, NY). Allelic discrimination real‐time PCR reactions were performed in a 384‐well‐plate format in duplicate for each individual sample according to manufacturer's instructions. Briefly, 20 ng of genomic DNA was mixed with 2.5 μL of 2× TaqMan^®^ Universal PCR Master Mix and 0.25 μL of 20× SNP Genotyping Assay in a total volume of 5 μL per well. Assays were run in an ABI 7900HT Fast Real‐Time PCR System (Thermo Fisher Scientific, Grand Island, NY). Allele calling was determined by end‐point fluorescent signal analysis using the ABI's SDS2.3 software (SABiosciences ‐ QIAGEN Inc., 27220, Turnberry Lane, Valencia, CA 91355).

### Quality control and filtering

GenomeStudio data were imported to SVS 7.6.7, Golden Helix's^®^ (Golden Helix, Inc. Bozeman, MT (http://www.goldenhelix.com), an integrated collection of analytic tools for managing, analyzing, and visualizing multifaceted genomic and phenotypic data. Parameters for excluding markers from analyses included: (1) deviations from the Hardy–Weinberg equilibrium with *P *<* *0.0000002 (0.05/250.000 markers) in both cases and controls, (2) a genotype call rate <90%, and (3) the presence of both more or less than two alleles. Genotype and allelic frequencies were estimated by maximum likelihood. Data subsets for common and rare variants where established using a minor allele frequency (MAF) criterion of 1.0% that was estimated from the whole sample data. Linkage disequilibrium (LD) pruning using CHM was applied to the subset of common markers using as parameters a window size of 50 markers with a window increment of 50 and an *r* ^2^ threshold of 0.5. With this pruned subset of common markers, matrices of identity by descent (IBD) and probability of identity were estimated. Those were used afterward for the mixed models analyses.

### Genetic analysis

Single allelic, additive, and dominant linear‐mixed effect models (LMEMs) (Liu and Leal 2010a,b; Segura et al. 2012) were fitted to test the association of these variants to ADHD. The advantage of these models is the inclusion of both fixed (sex and years of education, among other clinical variables) and random effects (the later to account for kinship effects by including the IBD matrix). The single‐locus LMEM assumes that all loci have a small effect on the trait, while multi‐locus LMEM assume that several loci have a large effect on the trait. In a single‐locus model, the association between the variant of interest and the disease trait is tested after covariates and genetic stratification are controlled for. Conversely, in a multi‐locus model the association is tested after covariates, genetic stratification and the effect of the remaining *m*‐1 variants are controlled for. These recently emerged methods have been proven to be more powerful than existing methods (Segura et al. 2012). Furthermore, this family of models allows handling of confounding effects and account for loci of small‐ and large‐effect in structured populations with a small computational burden (Segura et al. 2012). Variants significantly associated with the ADHD phenotype were determined after correction by multiple testing using the false discovery rate (FDR) (Benjamini and Hochberg 1995) and an alternative method based on extreme‐values theory (Vélez et al. 2014). As this is an attempt of evaluate an already known association, we have used the criterion of a *P*‐value ≤0.10 to consider a positive replication.

## Results

**Figure 1:**
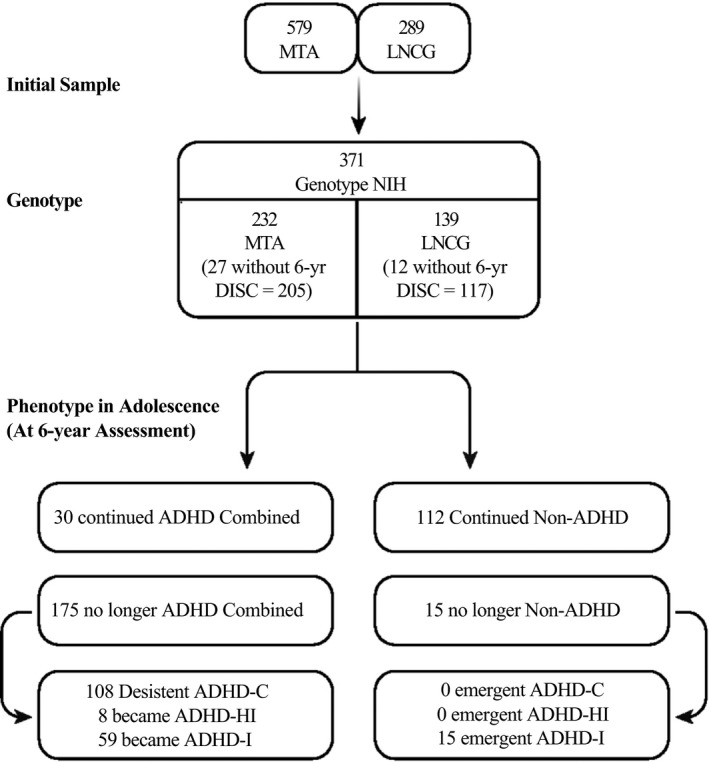
Diagram of individuals participation in the studies and collected DNA samples in relation of ADHD diagnosis.

A full description of demographic and clinical variables of the MTA has been published elsewhere (1999; Jensen et al. 2007; Molina et al. 2013, 2009; Molina and Pelham 2003). For a genetic add‐on we genotyped 371 volunteers (280 males, 75%), consisting of 232/579 = 40.0% of the MTA group and 139/289 = 48.1% of the LNCG. Diagnoses from the 6‐year DISC‐P were available for 205 of the MTA and 127 of the LNCG volunteers. For the 205 MTA participants in the genetic add‐on study, 30/205 (14.6%) retained the DISC‐P diagnosis of ADHD‐C in adolescence, but 175/205 (85.4%) did not, with switches in adolescence to ADHD‐Inattentive Type (ADHD‐I) for 59/205 (28.8%), ADHD‐Hyperactive Type (ADHD‐HI) for 8/205 = 3.9%, and no subtype of ADHD for 108/205 (52.7%). For the 127 LNCG participants in the genetic add‐on study, 112/127 (88.2%) remained undiagnosed in adolescence by the DISC‐P, but 15/127 (11.8%) did not, and all of these 15 were diagnosed with Adolescent ADHD‐I (see Fig. 1 and Table 1).

**Table 1:** Cross tabulation of ADHD combined type based on the diagnostic interview schedule for children–parent version (DISC‐P) administered at the 6‐year follow‐up assessment (PAD3Y) versus the diagnosis of ADHD at the baseline. The unknown category refers to those cases without PAD3Y criteria. The 112 LNCG are those that did not match ADHD criteria at the 6‐year follow‐up assessment, that is, 15 individuals

SJTYP	PAD3Y	PAD3Y	PAD3Y	Row
	Control	ADHD combined type	Unknown	Totals
MTA	175	30	27	232
LNCG	112	0	12	124
Totals	287	30	39	356

Using LMEMs we initially compared allelic, genotype, and haplotype data of MTA and LNCG at the base line diagnosis but found no significant differences. For the subgroup with persisting combined type ADHD, we found significant associations with *ADGRL3* for allelic and haplotype comparisons, when compared with the other adolescent subgroups (adolescent ADHD‐I, ADHD‐HI, or Non‐ADHD). The minor allele C at the marker rs2345039 was significant (*P* = 0.005) after corrections by multiple comparisons (*P*_FDR _= 0.04), which is the same marker reported to be significant in the original manuscript that described variants harbored in *ADGRL3*conferring susceptibility to ADHD by our group (Arcos‐Burgos et al. 2010). The haplotype analysis show that a haplotype involving rs2345039‐rs61747658‐rs1397548 (CAA) is significantly associated with a refined phenotype of adolescent‐persistent ADHD‐C when compared with the LNCG (*P* = 0.005, *P*_FDR _= 0.05; Odds ratio = 3.47, CI‐95% 1.37–8.74 (Table 2). This significant association, based on a comparison of the *n* = 30 cases with persistent ADHD‐C versus the LNCG, was independent of the fitted model, that is, allelic, genotype dominant, or genotype codominant, and in each case the association tolerated FDR correction (Table 3). We also compared the subgroup with persisting ADHD‐C with the subgroup consisting of the other subtypes representing partial manifestation of the full syndrome (ADHD‐I and ADHD‐HI), but we found no differences.

**Table 2:** Allelic association analysis of markers harbored in the *ADGRL3* (Accession number NC_000004.12, GRCh38.p2). An FDR correction was applied to account for multiple comparisons

	Chr	Position	Func	HGVS nomenclature	Corr/Trend *P*	Corr/Trend FDR	Odds ratio (minor allele)	OR lower confidence bound (minor)	OR upper confidence bound (minor)	Minor allele (test allele)	Minor allele frequency	Allele freq. (cases)	Allele freq. (controls)
rs2345039	chr4	62765029	intron	g.61899312G>C	0.005	0.044	2.225	1.262	3.922	C	0.437	0.625	0.428
rs11131347	chr4	62759269	intron	g.61893552C>T	0.040	0.181	1.761	1.021	3.039	A	0.485	0.617	0.477
rs6856328	chr4	62437317	intron	g.61571600C>G	0.122	0.367	2.694	0.730	9.937	C	0.027	0.050	0.019
rs61747658	chr4	62800623	missense	g.61934906A>G	0.390	0.878	0.000			G	0.011	0.000	0.012
rs2172802	chr4	62453208	intron	g.61587491A>G	0.437	0.787	1.248	0.713	2.184	G	0.295	0.350	0.301
rs35106420	chr4	62758490	missense	g.61892773G>A	0.468	0.702	0.000			A	0.011	0.000	0.009
rs6551665	chr4	62739540	intron	g.61873823G>A	0.542	0.696	1.188	0.683	2.066	G	0.404	0.446	0.404
rs1947274	chr4	62744239	intron	g.61878522C>A	0.596	0.670	1.162	0.668	2.020	C	0.411	0.446	0.410
rs1397548	chr4	62845489	coding‐synon	g.61979772A>G	0.908	0.908	0.967	0.546	1.712	A	0.324	0.317	0.324

**Table 3:** Haplotype association analysis of markers harbored in the *ADGRL3* (Accession number NC_000004.12, GRCh38.p2). An FDR correction was applied

First marker	Chr	Position	Ref allele NCBI	Obs	Func	Block #	Haplotype	EM cases freq	EM controls freq	Chi‐squared *P*	Chi‐squared FDR	Odds ratio	Odds ratio lower 95% CI	Odds ratio upper 95% CI
rs2345039	chr4	62765029	G	C/G	introit	7	CAA	0.12	0.04	0.01	0.06	3.47	1.37	8.78
rs35106420	chr4	62758490	G	A/G	Missense	5	GAC	0.62	0.42	0.01	0.08	2.22	1.26	3.91
rs11131347	chr4	62759269	C	C/T	intron	6	ACA	0.62	0.42	0.00	0.14	2.24	1.27	3.96
rs11131347	chr4	62759269	C	C/T	intron	6	GGA	0.37	0.51	0.04	0.20	0.56	0.32	0.99
rs1947274	chr4	62744239	C	A/C	intron	4	AGA	0.26	0.15	0.03	0.20	2.00	1.05	3.82
rs1947274	chr4	62744239	C	A/C	intron	4	AGG	0.30	0.44	0.04	0.21	0.54	0.30	0.98
rs35106420	chr4	62758490	G	A/G	Missense	5	GGG	0.37	0.52	0.03	0.25	0.54	0.31	0.95
rs11131347	chr4	62759269	C	C/T	intron	6	AGA	0.00	0.04	0.11	0.32	0.00	0.00	
rs2345039	chr4	62765029	G	C/G	intron	7	CAG	0.51	0.39	0.09	0.33	1.60	0.92	2.78
rs35106420	chr4	62758490	G	A/G	missense	5	GAG	0.00	0.04	0.11	0.34	0.00	0.00	
rs2345039	chr4	62765029	G	C/G	intron	7	GAG	0.17	0.28	0.09	0.36	0.54	0.26	1.11
rs2345039	chr4	62765029	G	C/G	intron	7	GAA	0.20	0.28	0.21	0.55	0.65	0.33	1.28
rs6856328	chr4	62437317	C	C/G	intron	1	GGG	0.19	0.14	0.26	0.59	1.50	0.74	3.04
rs6856328	chr4	62437317	C	C/G	intron	1	CAG	0.04	0.02	0.29	0.62	2.27	0.48	10.84
rs2345039	chr4	62765029	G	C/G	intron	7	GGG	0.00	0.01	0.39	0.63	0.00		
rs2172802	chr4	62453208	A	A/G	intron	2	GGC	0.19	0.14	0.26	0.63	1.50	0.74	3.05
rs11131347	chr4	62759269	C	C/T	intron	6	GGG	0.00	0.01	0.39	0.66	0.00		
rs61747658	chr4	62800623	A	A/G	missense	8	GG	0.00	0.01	0.39	0.69	0.00		
rs6856328	chr4	62437317	C	C/G	intron	1	GAA	0.37	0.44	0.35	0.71	0.77	0.43	1.35
rs2172802	chr4	62453208	A	A/G	intron	2	AAA	0.37	0.43	0.39	0.73	0.78	0.44	1.37
rs6551665	chr4	62739540	G	A/G	intron	3	AAG	0.55	0.58	0.60	0.80	0.86	0.50	1.50
rs6551665	chr4	62739540	G	A/G	intron	3	GCG	0.45	0.40	0.60	0.80	1.16	0.67	2.02
rs1947274	chr4	62744239	C	A/C	intron	4	CGA	0.37	0.32	0.53	0.80	1.20	0.68	2.13
rs6856328	chr4	62437317	C	C/G	intron	1	GGA	0.18	0.16	0.65	0.84	1.18	0.58	2.41
rs6856328	chr4	62437317	C	C/G	intron	1	GAG	0.22	0.25	0.58	0.84	0.83	0.43	1.61
rs2172802	chr4	62453208	A	A/G	intron	2	GAA	0.18	0.16	0.70	0.86	1.15	0.56	2.35
rs61747658	chr4	62800623	A	A/G	missense	8	AG	0.68	0.66	0.76	0.90	1.09	0.62	1.93
rs61747658	chr4	62800623	A	A/G	missense	8	AA	0.32	0.32	0.91	0.91	0.97	0.55	1.71
rs1947274	chr4	62744239	C	A/C	intron	4	CGG	0.08	0.09	0.83	0.91	0.89	0.32	2.48
rs2172802	chr4	62453208	A	A/G	intron	2	AGC	0.25	0.27	0.80	0.92	0.92	0.49	1.73
rs1397548	chr4	62845489	A	A/G	coding‐synon	9	G	0.68	0.68	0.91	0.94	1.03	0.58	1.83
rs1397548	chr4	62845489	A	A/G	coding‐synon	9	A	0.32	0.32	0.91	0.97	0.97	0.55	1.71

## Discussion

A large number of studies show that *ADGRL3* variants predispose to ADHD, modulate the pattern of brain metabolism, and predict ADHD severity (comorbidity with conduct disorder [CD], oppositional defiant disorder [ODD], and substance used disorder [SUD]) and response to stimulant medication (Arcos‐Burgos and Muenke 2010; Arcos‐Burgos et al. 2010, 2012; Acosta et al. 2011; Martinez et al. 2011; Ribases et al. 2011; Jain et al. 2012). Furthermore, *ADGRL3* susceptibility variants interact with haplotype variants harbored at chromosome 11q22 in the region of *NCAM1, TTC12, ANKK1,* and *DRD2* genes (Jain et al. 2012). It is clear now that the association between ADHD and *ADGRL3* variants identifies a subtype of the condition characterized by the presence of severe externalizing symptoms and consequently a complex prognosis of persistence and graveness.

In this manuscript, we present additional evidence that the *ADGRL3* variant rs2345039, previously reported to be associated with the ADHD susceptibility haplotype, predisposes to the development of a refined ADHD phenotype characterized by persistence of ADHD combined subtype symptoms into adolescence. The fact that we are detecting association of the *ADGRL3* variants in an extreme phenotype based on outcome of 6 years after childhood diagnosis – and confirmed by a rigorous prospective follow‐up – provides additional evidence that the association with *ADGRL3* not only is not spurious, but also is associated with a more severe form of ADHD.

These findings may partially explain the heterogeneity of longitudinal course in ADHD that includes differential symptom reduction with age, comorbidity, and ultimately, overall long‐term physical and mental health. Limitations of this study include the use of a final small sample size for those with persistent phenotype for ADHD at 6 years of age. However, it is known that in small sample size cohorts, where a high number of markers have been genotyped, major fluctuations in the distribution of significant values might appear and, consequently, spurious associations might reach significance. To control for this problem, we corrected by multiple comparisons using FDR as described in the Methods section. Furthermore, given that the direction of association between ADHD susceptibility and LPHN3 variants is well known, a *P*‐value of 0.10 was selected as the threshold to declare significance. The reason for selecting this threshold lies in the demonstration that for a given *P*‐value, the probability of replicating a significant result, under certain assumptions, is smaller than expected. Or better, as described by Goodman (Goodman 1992): “It is shown that if the observed difference is the true one, the probability of repeating a statistically significant result, the “replication probability”, is substantially lower than expected”.

Continued research on this genetic variant in samples followed into adulthood, and that include comprehensive assessment of symptoms and functioning, is warranted.

## Conflict of Interest

The authors declare no conflict of interests.
